# Evolution of Coronary Flow in an Experimental Slow Flow Model in Swines: Angiographic and Pathological Insights

**DOI:** 10.1155/2015/623986

**Published:** 2015-10-11

**Authors:** Yupeng Bai, Liqun Hu, Delong Yu, Sheng Peng, Xiaogang Liu, Mingjing Zhang, Ye Gu

**Affiliations:** Department of Cardiology, Wuhan Puai Hospital, Huazhong University of Science and Technology, Wuhan 430030, China

## Abstract

*Objective*. Pathomechanism of coronary slow flow phenomenon remains largely unclear now. Present study observed the pathological and angiographic evolution in a pig model of coronary slow flow. *Methods*. Coronary slow flow was induced by repeat coronary injection of small doses of 40 *µ*m microspheres in 18 male domestic pigs and angiographic and pathological changes were determined at 3 hours, 7 days, and 28 days after microspheres injection. *Results*. Compared to control group treated with coronary saline injection (*n* = 6) and baseline level, coronary flow was significantly reduced at 3 hours and 7 days but completely recovered at 28 days after coronary microsphere injection in slow flow group. Despite normal coronary flow at 28 days after microsphere injection, enhanced myocardial cytokine expression, left ventricular dysfunction, adverse remodelling, and ischemia/microembolism related pathological changes still persisted or even progressed from 3 hours to 28 days after coronary microsphere injection. *Conclusions*. Our results show that this large animal slow flow model could partly reflect the chronic angiographic, hemodynamic, and pathological changes of coronary slow flow and could be used to test new therapy strategies against the slow flow phenomenon.

## 1. Introduction

Coronary slow flow phenomenon (CSFP) is a disease entity referring to the angiographic observation of delayed contrast filling of the coronary vasculature in the absence of an anatomic obstruction during coronary angiography [1]. The reported incidence of CSFP was about 1%–7% in patients undergoing diagnostic angiography because of clinical suspicion of cardiovascular disease [2]. In a retrospective study using corrected TIMI frame count (CTFC) as an objective criterion of CTFC, as high as 25% of patients evaluated for typical angina or angina-like chest pain syndrome with normal epicardial coronary arteries were found to have CSFP [3]. It was shown that total cholesterol and LDL-C levels, body mass index, and incidence of metabolic syndrome and hospital admissions were all significantly higher in CSFP patients compared to control subjects [4]. CSFP is also related to poor outcome in patients treated with percutaneous transluminal coronary angioplasty (PCI) for first acute myocardial infarction (AMI) compared with PCI treated AMI patients without CSFP and in patients solely diagnosed with CSFP compared with patients without CSFP [5, 6]. The treatment modalities for CSFP are not well established till now due to the limited understanding on the pathogenesis of CSFP [4, 7]. Although the pathophysiological mechanisms of CSFP remain uncertain [8], coronary microembolism serves as an important mechanism for the development and progression of CSFP [9]. Animal models mimicking clinical CSFP are valuable tools for exploring the pathomechanisms and new therapy strategies. We previously established a coronary thrombotic microembolism model in rats [10]. A swine model with angiographic CSFP was newly established by repeat coronary injection of small doses of 40 *μ*m microspheres in our laboratory [11]. In the present study, we observed the pathological and angiographic evolution in this CSFP model.

## 2. Materials and Methods 

### 2.1. Animal

Male domestic pigs (*n* = 24, 3 to 4 months old, 25 ± 2 kg) were used in this study. Aspirin (2-3 mg/kg/d) was mixed in the food 3 days prior to experimental studies. Study protocol was approved by the Tongji Medical College Council on the Animal Care Committee of Huazhong University of Science and Technology (Wuhan, China). Animals were maintained in accordance with the Guide for the Care and Use of Laboratory Animals published by the US National Institute of Health (NIH Publication Number 85-23, revised 1996). Animals were randomly assigned to slow flow groups (SF, *n* = 18) and sham-operated group (CON, *n* = 6).

### 2.2. Surgery

The pigs were fasted for 12 hours before operation and had free access to drinking water until 4 hours prior to operation. Pigs were anesthetized by an intramuscular injection of ketamine (15 mg/kg) combining with atropine (1 mg) and then fixed in a supine position on the workstation. During the operation, 3–5 mL 3% pentobarbital sodium solution was injected via ear marginal vein on demand to maintain the anaesthesia state. ECG and vital signs were continuously monitored. Oxygen saturation (SO_2_) was measured with pulse oximeter attached on the ear of pigs. Anticoagulation was induced with 200 IU/kg heparin sodium. The right femoral artery was dissected and 6F vascular sheath was placed for arterial access. After initial coronary angiography (CAG) using 6F JR3.5 guiding catheter (Medtronic, Inc.), ventriculography examinations, and LV pressure measurements with 5F Pigtail catheter (Cordis Inc.), a 2.6F infusion microtubule catheter (Terumo Corporation) was advanced to the middle part of the left anterior artery (LAD, around 30 mm from the LAD orifice) with the help of the guide wire (Whisper, Abbott Vascular) for microspheres or saline injection. Pigs in CON group received repeated equal volume coronary saline injection as in the SF group (see below). Stock solution which contains 120000 40 *μ*m microspheres/mL (NT33N, Bangs Laboratories, Inc.) was prepared, and 0.1 mL stock solution containing 12000 microspheres was diluted into 5 mL saline and injected through the 2.6F infusion microtubule catheter for 20 seconds. Five minutes later, 0.2 mL stock solution containing 24000 microspheres was diluted into 5 mL saline and injected for 20 seconds; this procedure was repeated 5 minutes later, and then 0.3 mL stock solution containing 36000 microspheres was diluted into 5 mL saline and injected for 20 seconds through the 2.6F infusion microtubule catheter for pigs assigned to SF group in 5 minutes' interval repeatedly till the appearance of angiographic slow flow as defined by TIMI frame count (TFC) > 40. The blood flow in the LAD was assessed with the TIMI scale and TFC, while myocardial tissue perfusion was analyzed with the TMPG scale (TIMI myocardial perfusion grade) [12]. Coronary angiography (CAG) imaging was measured at baseline, immediately at the time of slow flow formation, 5 minutes, 15 minutes, 30 minutes, 1 hour, and 3 hours after microspheres injection. Left ventricular (LV) end-diastolic volume and ejection fraction (LVEF) as well as LV pressure were assessed at baseline and 3 hours after microspheres injection. Six animals were sacrificed by intravenous 10% KCl injection (30 mL) to stop the heart at diastolic phase under deep anaesthesia (intramuscular injection of 75 mg/kg ketamine) for biochemical and pathological analysis at 3 hours after coronary microsphere injection (SF-3H). Another 6 animals were sacrificed by intravenous 10% KCl injection (30 mL) to stop the heart at diastolic phase under deep anaesthesia (intramuscular injection of 75 mg/kg ketamine) for biochemical and pathological analysis at 7 days after coronary microsphere injection (SF-7D). The remaining 5 animals (one pig died) in SF group (SF-28D) and 6 animals in CON group were reanesthetized at 7 days and 28 days after procedure for CAG and ventriculography examinations, respectively. Prophylactic antibiotics (50 mg/kg cephalothin (IV) and 5 mg/kg gentamicin (IM)) were administered for 3 days for animals not sacrificed at 3 hours after coronary microsphere or saline injection. Butorphanol (0.025 mg/kg) was administered intramuscularly after operation as required to alleviate pain.

### 2.3. Serum Markers

Blood (10 mL) was collected via femoral vein at baseline, 3 hours, 24 hours, 7 days, and 28 days after microsphere injection from animals in the CON group and in the SF-28D group and centrifuged and serum was stored at −80°C. Cardiac troponin I (c-TNI), von Willebrand factor (vWF), and endothelin-1 (ET-1) levels were determined by respective ELASA kits (Wuhan Elabscience Biotechnology Co., Ltd.) according to the manufacturer's instructions.

### 2.4. Pathologic Examination

#### 2.4.1. Light Microscopic Analysis

At 28 days after microsphere or saline injection, 6 pigs in the CON group and 5 pigs in the SF-28D group were sacrificed by intravenous 10% KCl injection (30 mL) to stop the heart at diastolic phase under deep anaesthesia (intramuscular injection of 75 mg/kg ketamine). For pigs sacrificed at 3 hours, 7 days, and 28 days after coronary injection, heart was excised, right and left atria were removed, and left ventricle (after separating the right ventricle) was washed with saline and weighed. Left ventricular (LV) apex was cut for Western blot analysis (see below) and LV free wall near LV apex was cut into 0.5 cm × 0.5 cm block and fixed in 10% formalin for three days and then it was dehydrated, paraffin-embedded, and cut into 4 *μ*m sections for hematoxylin and eosin (HE), hematoxylin-basic fuchsin-picric acid (HBFP), and Carstairs staining. Van Gieson's staining and smooth muscle motor protein (Smooth Muscle Actin, SMA; GM85110) staining were also performed on myocardial tissue derived from LV free wall. For each animal, 10 random pictures were obtained at 200x magnification and the results were analyzed by a microscope connected with computer and microscopic image analysis system (Image Pro-4, Media Cybernetics, Inc., Atlanta, GA). To observe the microthrombosis in the coronary arteriole, 5 sections were observed on each animal, 4 pictures from each section were analyzed, and 5 arterioles with diameters less than 200 *μ*m were counted and evaluated from each picture; thus, 100 coronary arterioles with diameters less than 200 *μ*m from each animal were observed under microscope (magnification, ×100) in HE-stained sections and the percentage of microthrombosis was determined. To analyze the inflammatory response, the inflammatory cells (leukomonocytes) were counted totally in 10 randomly selected visual fields in each section and five sections per pig were examined.

### 2.5. Western Blot Analysis

Protein expression of tumor necrosis factor-alpha (TNF-*α*) and interleukin-6 (IL-6) on left ventricular apex obtained from sacrificed animals at 3 hours, 7 days, and 28 days after microsphere injection and 28 days after saline injection was determined by Western blot analysis. Tissues were homogenized in PBS and centrifuged at 10,000 ×g for 10 minutes at 4°C, and then 70 *μ*g supernatant was lysed in electrophoresis buffer, boiled for 10 minutes, and subsequently subjected to electrophoresis on a SDS-polyacrylamide gel. The separated blots were transferred to nitrocellulose membranes and blocked for 1 hour in TTBS buffer containing 5% nonfat milk. The membranes were incubated overnight with primary TNF-alpha (Abcam Co. ab1793) or IL-6 (Bioworld Co., BS6419) polyclonal antibodies (TNF-*α*, 1 : 500 dilution; IL-6, 1 : 200 dilution) and then with horseradish peroxidase- (HRP-) conjugated goat anti-rabbit IgG antibody (1 : 1,000 dilution) for 2 hours at 37°C. Blots were detected by chemiluminescence and relative protein expression was quantified by scanning densitometry.

### 2.6. Statistical Analyses

The data were analyzed using SPSS 12.0 (SPSS, Inc., Chicago, IL, USA). Data are presented as the mean ± SD. Differences in mean values between the 4 groups and between SF and CON groups were compared by one-way ANOVA followed by Student-Newman-Keuls post hoc test or unpaired Student's *t*-test, as indicated. *P* < 0.05 was considered statistically significant.

## 3. Results

### 3.1. Mortality

One pig died at 2 days after microsphere injection, and postmortem examination revealed pulmonary edema and massive respiratory secretions in this pig. The remaining 23 pigs survived to the designed study end.

### 3.2. Left Ventricular Weight and Left Ventricular Weight/Body Weight Ratio

As shown in Table 1, LV/BW ratio was significantly higher in 7D-SF and 28D-SF groups compared to CON group.

### 3.3. Blood Pressure Change after Coronary Saline or Microsphere Injection

Femoral arterial blood pressure was not affected by coronary saline injection, which was significantly reduced immediately after each coronary microsphere injection and then recovered at 5 minutes but still lower than baseline level and femoral arterial blood pressure returned to baseline level at 7 days and 28 days after coronary microsphere injection.

### 3.4. Evolution of Coronary Microsphere Injection Induced Coronary Slow Flow

**Figure 1:**
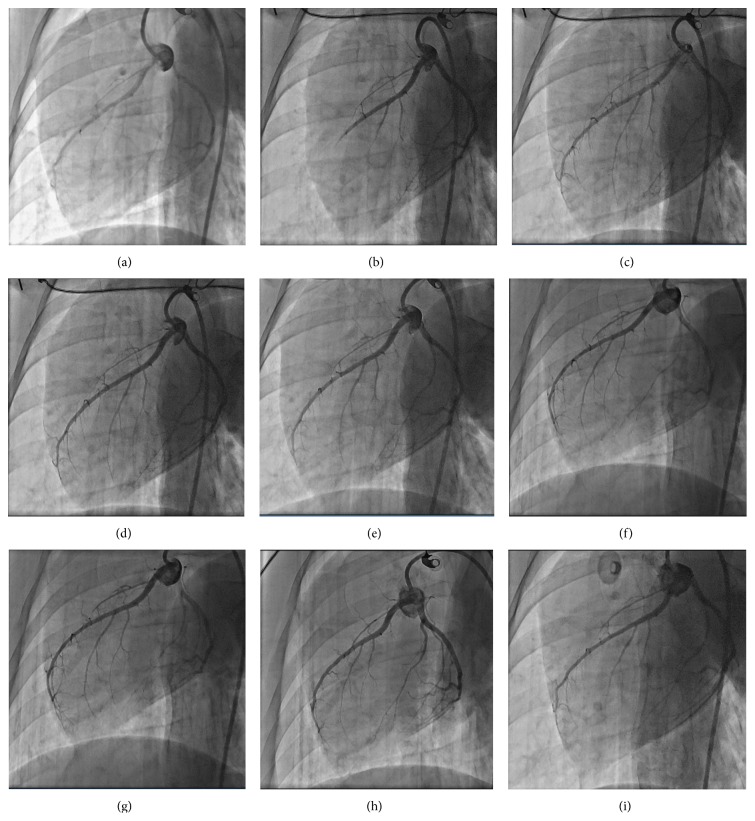
(a–i) Representative coronary angiography (CAG) imaging at baseline (a, TIMI grade 3), immediately at the time of slow flow formation (b, TIMI grade 2), 5 min (c, TIMI grade 2), 15 min (d, TIMI grade 2), 30 min (e, TIMI grade 2), 1 h (f, TIMI grade 2), 3 h (g, TIMI grade 2), 7 days (h, TIMI grade 2), and 28 days (i, TIMI grade 3) after microsphere injection.

**Figure 2:**
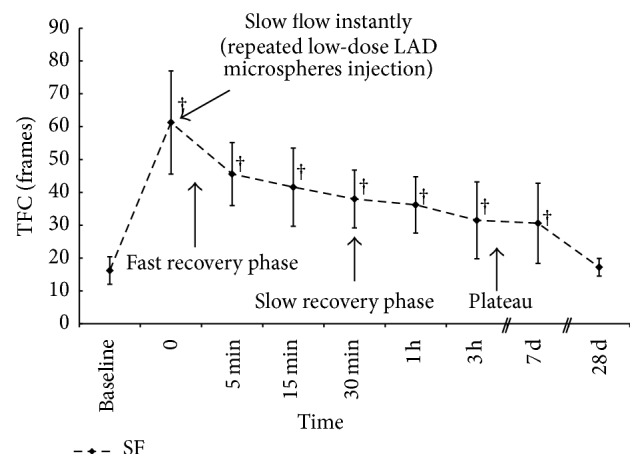
Representative CAG imaging TFC at baseline, immediately at the time of slow flow formation, 5 minutes, 15 minutes, 30 minutes, 1 hour, 3 hours, 7 days, and 28 days after coronary microspheres injection. ^†^ *P* < 0.05 versus baseline.

The mean injected stock solution was 2.45 ± 0.52 mL (around 294000 microspheres) to induce coronary slow flow in the pigs.  Figure 1 and Supplemental video files in Supplementary Material available online at http://dx.doi.org/10.1155/2015/623986 show representative CAG imaging at baseline, immediately at the time of slow flow formation, 5 minutes, 15 minutes, 30 minutes, 1 hour, 3 hours, 7 days, and 28 days after microspheres injection. Coronary flow presented fast recovery, slow recovery, and plateau period after repeat coronary microsphere injection and remained reduced till 7 days after microsphere injection and returned to baseline level at 28 days after microsphere injection as evidenced by TFC (Figure 2). Similarly, TIMI and TMPG were significantly reduced at 3 hours and 7 days after coronary microsphere injection and returned to baseline level at 28 days after coronary microsphere injection (Table 2). Coronary flow was not affected by coronary saline injection.

Compared to baseline values and CON group, heart rate was significantly reduced at 3 hours after microsphere injection and returned to baseline level at 7 and 28 days after microsphere injection. LVSP and LVEF were significantly reduced, while LVEDP was significantly increased at 3 hours, 7 days, and 28 days after microsphere injection (Table 2, all *P* < 0.05). LV end-diastolic and end-systolic volumes were significantly increased at 28 days after microsphere injection. Thus, repeat coronary microsphere induced progressive LV dysfunction and adverse LV remodelling, while coronary flow was recovered at 28 days after microsphere injection.

### 3.5. Serial Changes of Serum c-TNI, vWF, and ET-1

As shown in Table 3, serum c-TNI value peaked at 24 hours and remained elevated at 7 days and was slightly higher at 28 days after microsphere injection. vWF is an important parameter reflecting endothelial dysfunction and blood coagulation activation [13]. vWF peaked at 3 hours and remained significantly increased at 7 days and was still slightly higher at 28 days after microsphere injection. Endothelin-1 (ET-1) is vasoconstriction peptide produced by the vascular endothelium; increased ET-1 was detected in case of myocardial ischemia and myocardial infarction [14]. Serum ET-1 level was significantly increased at 3 hours and peaked at 24 hours and remained significantly increased at 7 days and was still slightly increased at 28 days after microsphere injection compared to baseline value and CON group at respective time points after microsphere injection (Table 3).

### 3.6. Pathological Examinations

#### 3.6.1. HBPF Staining

**Figure 3:**
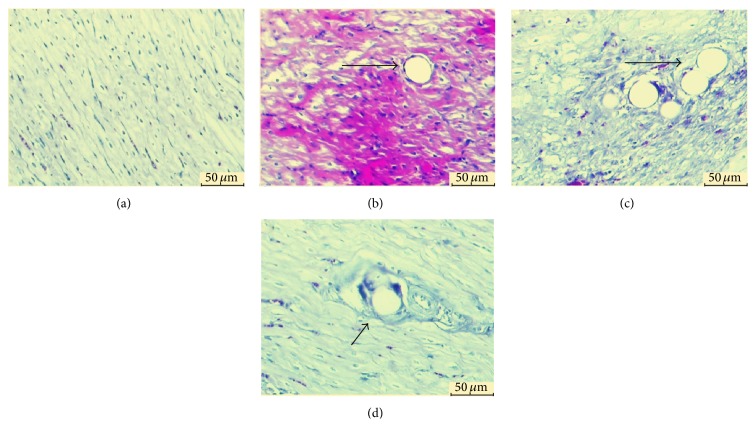
HBFP dyeing analysis: (a) CON group (normal myocardium, ×200); (b) 3H-SF group (red or purple stained ischemic myocardium around microsphere obstructed arteriole, ×200); (c) SF-7D group (microsphere obstructed arteriole and surrounding myocardium without ischemia, ×200); (d) SF-28D group (microsphere and surrounding arteriole and nonischemic myocardium, ×200). Arrows indicate microsphere obstructed arteriole.

Early myocardial ischemia changes could be detected by HBPF staining: normal myocardium stained yellow, while ischemic tissue stained red or purple [15]. Myocardial ischemic changes were only detected in SF-3H group (Figure 3(b)). The ischemic area (IA) was calculated using the following formula: IA  (%) = IA/area  of  field  of  whole  vision × 100. IA was 7.9 ± 1.4% in the SF-3H group (Figure 3).

#### 3.6.2. HE Staining

**Figure 4:**
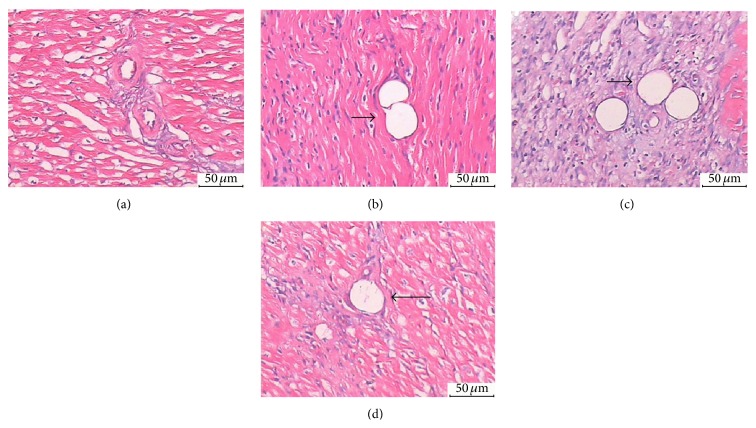
HE dyeing analysis: (a) CON group (arteriole, ×200); (b) SF-3H group (microsphere obstructed arteriole, ×200); (c) SF-7D group (microsphere obstructed arteriole and surrounding microinfarction area, ×200); (d) SF-28D group (microsphere obstructed arteriole and surrounding microinfarction area, ×200). Arrows indicate microsphere obstructed arteriole.

Microthrombosis in the coronary arteriole was examined in the HE-stained heart sections. Microthrombosis (Figure 4) was similar among the 3 SF groups (24.5 ± 4.37% in the SF-3H group, 24.5 ± 6.3% in the SF-7D group, and 24.0 ± 6.4% in the SF-28D group, *P* > 0.05).

#### 3.6.3. Inflammatory Cell Infiltration after Coronary Microsphere Injection

**Figure 5:**
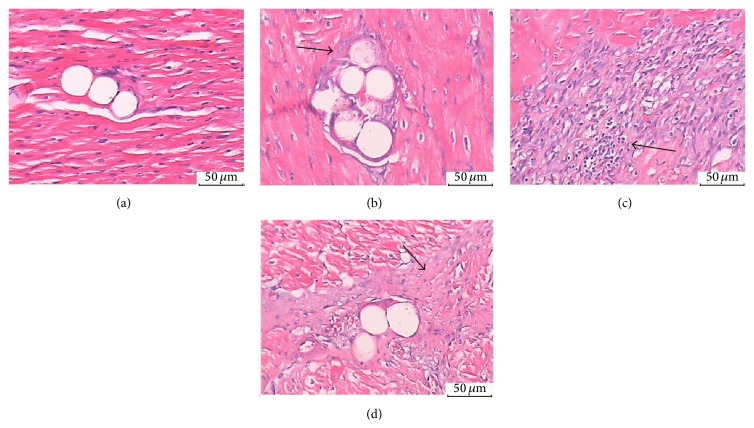
Inflammatory cell infiltration analysis: (a) SF-3H group (microsphere obstructed arteriole, ×200); (b) SF-7D group (microspheres obstructed arteriole, ×200); (c) SF-7D group (around the microinfarction area, ×200); (d) SF-28D group (microsphere obstructed arteriole and surrounding microinfarction area, ×200).

Inflammatory cell infiltration was observed on HE-stained heart sections. Inflammatory response was not visualized in heart of SF-3H and SF-28D groups (Figures 5(a) and 5(d)). Multinucleated giant cells were found in microspheres obstructed arteriole (Figure 5(b), arrow), and coagulative necrosis and fibroblast proliferation were seen in the microinfarct zone and leukomonocyte infiltration was observed around the microsphere obstructed vessel (Figure 5(c), arrow) in the heart of SF-7D group. There were 745 ± 163 leukomonocyte cell infiltration in the SF-7D group. Collagen deposition was evidenced in the heart at 28 days after microsphere injection (Figure 5(d)).

#### 3.6.4. CARSTAIRS Staining

**Figure 6:**
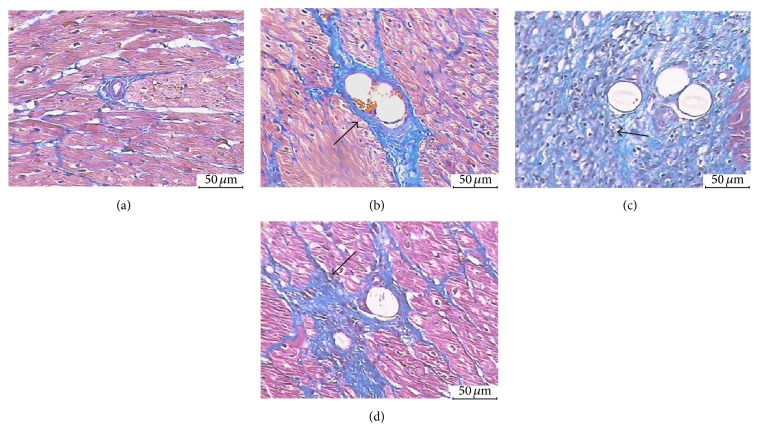
CARSTAIRS staining analysis: (a) CON group (arteriole, ×200); (b) SF-3H group (microsphere obstructed arteriole, ×200); (c) SF-7D group (microsphere obstructed arteriole and surrounding microinfarction area, ×200); (d) SF-28D group (microsphere obstructed arteriole and surrounding microinfarction area, ×200).

CARSTAIRS staining was performed to distinguish micro thromboembolism ingredients. Myocardial arterial thrombosis and platelet thrombus stained grey blue to light blue; red blood cells stained yellow or orange; fibrin stained bright red; myocardial tissue stained red; collagen fibers stained blue; and fibroblast stained sapphirine. Microthrombosis was visualized in the SF-3H group, and microthrombosis was composited mainly with fibrin, platelets, and some of the red blood cell aggregates (Figure 6(b), arrow). Fibroblast proliferation was observed in the SF-7D group (Figure 6(c), arrow) and collagen was seen in the SF-28D group (Figure 6(d), arrow).

#### 3.6.5. Van Gieson's Staining

**Figure 7:**
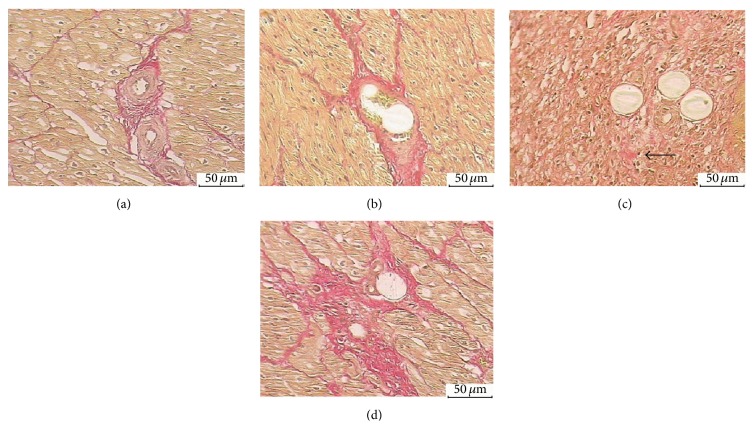
VG staining analysis: (a) CON group (arteriole, ×200); (b) SF-3H group (microsphere obstructed arteriole, ×200); (c) SF-7D group (microsphere obstructed arteriole and surrounding microinfarction area, ×200); (d) SF-28D group (microsphere obstructed arteriole and surrounding microinfarction area, ×200).

**Figure 8:**
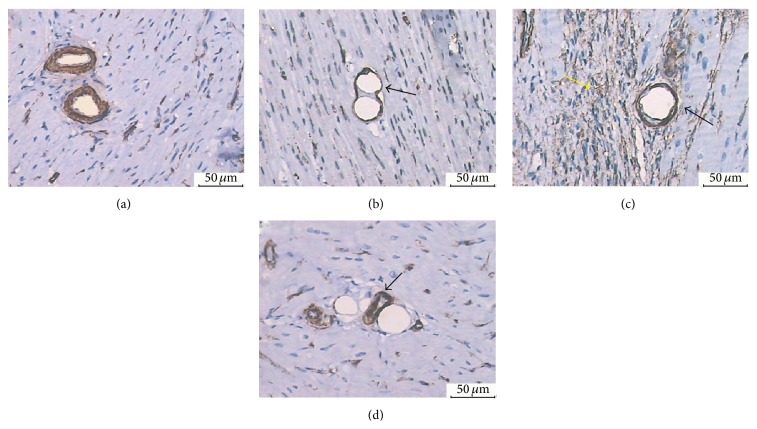
SMA immunohistochemistry analysis: (a) CON group (arteriole, ×200); (b) SF-3H group (microspheres obstructed arteriole ×200); (c) SF-7D group (microsphere obstructed arteriole and surrounding microinfarction area, ×200); (d) SF-28D group (microspheres and surrounding arteriole, ×200).

Van Gieson's (VG) staining was performed to quantify collagen and cardiomyocytes stained yellow and collagen fibers stained red. Myocardial collagen volume fraction (CVF) was calculated as follows: myocardial collagen area/total area × 100%. CVF was significantly increased in the SF-28D group (5.92 ± 1.39%, Figure 7(d)) compared with the CON group (1.22 ± 0.52%, Figure 8(a)), SF-3H group (1.25 ± 0.59%, Figure 7(b)), and SF-7D group (1.33 ± 0.52%, Figure 7(c)). Fibroblast proliferation and small amount of collagen fibers were also observed at microinfarct zone in the SF-7D group (Figure 7(c)).

### 3.7. Immunohistochemistry

SMA staining was performed to see if microspheres were located in the vascular lumen or not under light microscope. Microspheres were not detected in microvascular vessels in CON group (Figure 8(a)) but were detected in the SF-3H group (Figure 8(b), black arrow) and SF-7D group (Figure 8(c), black arrow); proliferation of vascular network near the microspheres was also detected in the SF-7D group (Figure 8(c), yellow arrow). Immunohistochemical staining also evidenced microspheres outside microvascular lumen in SF-28D group (Figure 8(d), black arrow).

### 3.8. Western Blot Analysis

**Figure 9:**
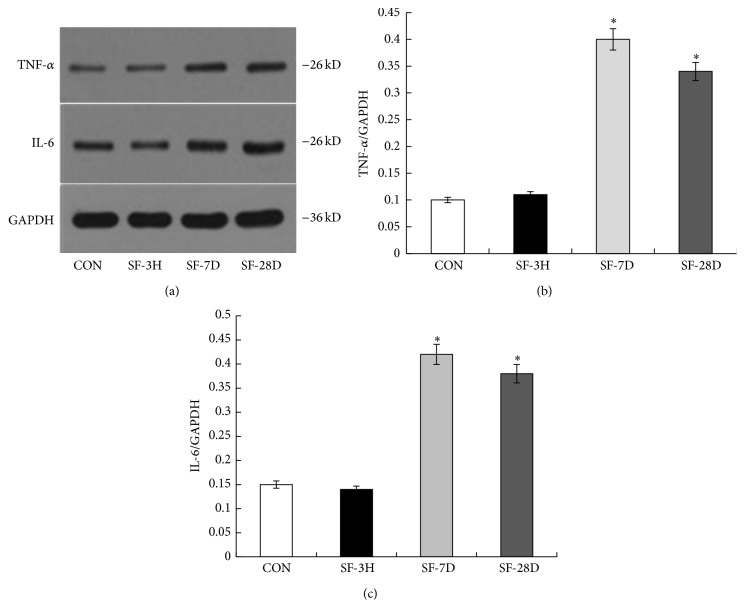
Myocardial protein expression of TNF-*α* and IL-6. (a) Western blots. (b) Barographs of TNF-*α*/GAPDH ratio. (c) Barographs of IL-6/GAPDH ratio. ^*∗*^ *P* < 0.01 versus CON.

As shown in Figure 9, myocardial protein expression of inflammatory cytokines IL-6 and TNF-*α* was significantly upregulated in the SF-7D and SF-28D groups compared to CON group.

## 4. Discussion

The major findings of present study are as follows. (1) Repeat small doses coronary microsphere injection induced myocardial pathological changes were similar to those induced by coronary ischemia/reperfusion injuries. (2) Despite recovered coronary flow at 28 days after microsphere injection, left ventricular dysfunction and adverse LV remodelling progressed during the observation period in this slow flow model in pigs. Thus, this large animal model partly reflects the angiographic and pathological changes of slow flow and could be used to evaluate new therapeutic strategies against slow flow phenomenon.

### 4.1. Coronary Flow and LV Function and Remodelling Evolution after Coronary Microsphere Injection

Previously, Skyschally et al. demonstrated decreased systolic wall thickening by 50% and reduced coronary and inotropic reserve but the coronary blood flow remained unchanged after stepwise repetitive microsphere (42 *μ*m: 158,000 ± 48,000) injection into the left circumflex coronary artery of dogs [16]; this study thus demonstrated the perfusion-contraction mismatch in microembolized myocardium. Angiographic evidence of slow flow was not known in above study. In our study, around 294000 microspheres with 40 *μ*m diameter were injected to LAD to induce slow flow in this pig model, so the amount of microsphere injected was larger in this study. In another study, Ma and colleagues injected 120000 42 *μ*m dynospheres into LAD of mini pig and LVEF was reduced at 6 hours and recovered at 7 days after injection, while LV remodelling changes continued and TIMI grade 3 coronary flow was not affected in this model [17]. Our results demonstrated reversible coronary flow but continued LV dysfunction and remodelling in this slow flow pig model. Thus, reduced coronary and inotropic reserve and reversible myocardial dysfunction and persistent remodelling could be achieved by smaller amount of coronary microsphere injection, while certain amount of coronary microembolism is essential to induce slow flow phenomenon in large animals; this is related to reversible coronary blood flow change but persistent LV dysfunction and remodelling.

Inflammation plays a crucial role in the pathogenesis of coronary microembolism [18]. Consistent with previous findings [19], increased inflammatory responses during the early course of microembolism and persistent upregulated proinflammatory cytokines protein expression and consequent collagen deposition were observed in this model, indicating the fundamental role of inflammation in the pathogenesis of slow flow.

### 4.2. Histopathological Evolution after Coronary Microsphere Injection

Histopathological examinations evidenced single microsphere or clumps of microspheres as well as platelet fibrin thrombi formation in myocardial vascular lumen at 3 hours after microsphere injection. Thus, coronary microsphere injection not only resulted in the obstruction of coronary microcirculation but also triggered in situ myocardial platelet fibrin thrombosis. These pathological changes might therefore be responsible for the observed myocardial ischemia and slow blood flow. Increased serum von Willebrand factor (vWF) and ET-1 levels at 3 hours and 24 hours after microsphere injection also indicated myocardial microvascular endothelial cells damage and platelet aggregation. Thus, besides the mechanical vascular obstruction, coronary microsphere injection also induced endothelial cell injury and dysfunction [20]. Early myocardial ischemia as evidenced by HBFP staining at 3 hours after microsphere injection was also reflected by significantly increased plasma c-TNI levels at this time point. c-TNI level was slightly higher at 28 days after coronary microsphere injection; this might be attributable to the LV dysfunction and failure at this stage [21]. Multinucleated giant cells were found in microspheres obstructed arteriole pathological changes at 7 days following coronary microsphere injection; this pathological finding is usually seen in chronic granulomatous disease and it is not a common finding in myocardium after ischemic insult; the underlying mechanism of the appearance of multinucleated giant cells after coronary microsphere injection warrants future studies. LV remodeling and collagen changes in this model resemble similar pathological changes after ischemic insult indicating the common threads of slow flow and myocardial ischemic injury [22].

Our data suggest the irreversible feature of slow flow after coronary microsphere induced microthrombosis and embolism. Previous studies demonstrated that smaller amount of coronary microsphere injection could decrease systolic wall thickening and reduce coronary and inotropic reserve and induce reversible LV systolic dysfunction and persistent LV remodelling. Our results showed that once slow flow is formed the left ventricular dysfunction and adverse remodelling as well as the enhanced inflammatory changes persisted and even progressed in this model during the 4 weeks observation period, despite the recovery of coronary blood flow at 28 days after coronary microsphere injection. These results could partly explain the worse outcome of slow flow patients since these patients are continuously facing the negative impacts of hemodynamic and pathological changes during the slow flow disease course. 

*Limitation*. The underlying pathological mechanisms of CSFP are multiple, including endothelial abnormality. Present slow flow model is induced in young swine by repeatedly injecting the microspheres into the coronary until the blood flow is slowed; it is quite different from the real clinic situation in that almost all patients with slow flow also have multiple risk factors for long time; present model could not mimic the completed pathology in the patients. Moreover, microspheres are chemically inert and not chemoattractant, so present slow flow model induced by repeatedly injecting the microspheres into the coronary in young swine is quite different from the clinical coronary slow flow induced by microemboli of atherosclerotic and thrombotic debris. In conclusion, this model partly reflects pathologic and angiographic changes of slow flow. This slow flow pig model might be a useful tool to test new therapy strategies against slow flow phenomenon.

## Supplementary Material

The short description (in paragraph style) of the Supplementary Material:
The measurement of TIMI flow grade and the TFC (TIMI flow count) was acquired by two experienced technicians. Each frame was recorded and analyzed by TOSHIBA Infinix cardiovascular interventional imaging system. Film speed was 30 frames per second. The following three criteria were used to define the first frame: (1) Contrast agent reached initiating terminal of the arteries; (2) Contrast agents contacted the two sides of initiating terminal of the artery; (3) Evidence of forward movement of the contrast agent. The last frame was counted when the contrast agent reached to the farthest portion of the branch, but there is no need to fulfill the branch. Anatomical marks: the farthest branch of anterior descending branch (LAD) was defined by the tail of the whale or grass fork imaging sign.
video 1: Representative CAG imagines showing TFC at baseline in a pig from SF-28D group (TIMI grade=3, TFC=15, corresponding Figure 1A).
video 2: Representative CAG imagines showing TFC immediately at the time of slow flow formation in a pig from SF-28D group (Coronary flow in LAD was very slow, TIMI grade=2, TFC=60, corresponding Figure 1B).
video 3: Representative CAG imagines showing TFC at 5min post slow flow formation in a pig from SF-28D group (Coronary flow in LAD presented fast recovery, TIMI grade=2, TFC=43, corresponding Figure 1C).
video 4: Representative CAG imagines showing TFC at 15min post slow flow formation in a pig from SF-28D group (Coronary flow in LAD presented slow recovery, TIMI grade=2, TFC=38, corresponding Figure 1D).
video 5: Representative CAG imagines showing TFC at 30min post slow flow formation in a pig from SF-28D group (Coronary flow in LAD presented slow recovery, TIMI grade=2, TFC=36, corresponding Figure 1E).
video 6: Representative CAG imagines showing TFC at 1 hour post slow flow formation in a pig from SF-28D group (Coronary flow in LAD presented slow recovery, TIMI grade=2, TFC=35, corresponding Figure 1F).
video 7: Representative CAG imagines showing TFC at 3 hours post slow flow formation in pig from SF-28D group (Coronary flow in LAD presented slow recovery, TIMI grade=2, TFC=29 , corresponding Figure 1G).
video 8: Representative CAG imagines showing TFC at 7 days post slow flow formation in a pig from SF-28D group (Coronary flow in LAD presented plateau period, TIMI grade=2, TFC=28, corresponding Figure 1H)
video 9: Representative CAG imagines showing TFC at 28 days post slow flow formation in a pig from SF-28D group (Coronary flow in LAD returned to baseline level, TIMI grade=3, TFC=16, corresponding Figure 1I).

## Figures and Tables

**Table 1:** Left ventricular (LV) weight and LV/body weight ratio.

Groups	CON	SF-3H	SF-7D	SF-28D
	(*n* = 6)	(*n* = 6)	(*n* = 6)	(*n* = 5)
LV (g)	150.0 ± 7.4	117.3 ± 7.3^*∗*^	123.2 ± 6.0^*∗*^	160.8 ± 5.8^*∗*^
BW (kg)	36.0 ± 2.0	25.5 ± 1.2^*∗*^	25.8 ± 1.1^*∗*^	33.2 ± 2.0^*∗*^
LV/BW (g/g, %)	0.42 ± 0.03	0.46 ± 0.02	0.48 ± 0.02^*∗*^	0.49 ± 0.01^*∗*^

LV, left ventricular weight; BW, body weight. All data showed with mean ± standard deviation. ^*∗*^ *P* < 0.05 versus CON group. Values of CON group derived at 28 days after saline injection.

**Table 2:** Angiographic and hemodynamic parameters at baseline, 3 hours, 7 days, and 28 days after LAD coronary microspheres injection.

Group	Variable	Time points			
		Baseline	3 hours	7 days	28 days
CON (*n* = 6)	HR (beats/min)	133 ± 13	122 ± 15^*∗*^	131 ± 8	129 ± 9
	TIMI (grade)	3 ± 0	3 ± 0	3 ± 0	3 ± 0
	TMPG (grade)	3 ± 0	3 ± 0	3 ± 0	3 ± 0
	TFC (frame)	15.8 ± 3.4	15.8 ± 1.7	16.2 ± 2.2	16.0 ± 2.4
	LVSP (mmHg)	150.6 ± 12.3	145.3 ± 15.8	147.8 ± 10.2	146.8 ± 8.2
	LVEDP (mmHg)	7.9 ± 2.4	8.6 ± 1.9	7.7 ± 2.4	7.7 ± 2.3
	LVEDV (mL)	24.0 ± 3.5	24.1 ± 3.6	24.8 ± 4.0	28.9 ± 4.2^*∗*^
	LVESV (mL)	7.7 ± 1.3	7.9 ± 1.4	8.2 ± 1.7	9.9 ± 1.5^*∗*^
	LVEF (%)	67.5 ± 3.8	66.8 ± 3.4	66.7 ± 3.9	65.4 ± 4.5

SF-28D (*n* = 5)	HR (beats/min)	129 ± 11	110 ± 7^*∗*†^	127 ± 7.3^†^	124 ± 6^†^
	TIMI (grade)	3 ± 0	2 ± 0^*∗*†^	2 ± 0^*∗*†^	3 ± 0
	TMPG (grade)	3 ± 0	0.3 ± 0.5^*∗*†^	0.8 ± 0.4^*∗*†^	3 ± 0
	TFC (frame)	16.2 ± 4.2	31.5 ± 11.7^*∗*†^	30.6 ± 12.2^*∗*†^	17.4 ± 2.7
	LVSP (mmHg)	146.8 ± 15.3	112.5 ± 10.3^*∗*†^	122 ± 8.2^*∗*†^	123.6 ± 8.9^*∗*†^
	LVEDP (mmHg)	7.7 ± 3.7	15.0 ± 2.1^*∗*†^	14.4 ± 1.5^*∗*†^	14.3 ± 1.2^*∗*†^
	LVEDV (mL)	24.5 ± 3.4	25.5 ± 3.7	28.7 ± 3.1^*∗*†^	33.0 ± 3.8^*∗*†^
	LVESV (mL)	7.7 ± 1.5	11.7 ± 2.6^*∗*†^	13.2 ± 2.5^*∗*†^	13.9 ± 3.1^*∗*†^
	LVEF (%)	68 ± 5	55 ± 5^*∗*†^	54 ± 8^*∗*†^	58 ± 6^*∗*†^

TIMI: thrombolysis in myocardial infarction, TMPG: TIMI myocardial perfusion grade, TFC: TIMI frame count, LVSP: LV systolic pressure, LVEDP: LV end-diastolic pressure, LVEDV: LV end-diastolic volume, LVESV (mL): LV end-systolic volume, and LVEF: LV ejection fraction. All the data showed with mean ± standard deviation. ^*∗*^ *P* < 0.05 versus baseline, ^†^ *P* < 0.05 versus CON group.

**Table 3:** Serum c-TNI, vWF, and ET-1 at different time points in CON group and SF group (SF-28D).

Groups	Variable	Time points				
		Baseline	3 hours	24 hours	7 days	28 days
CON (*n* = 6)	vWF (ng/mL)	1.10 ± 0.18	1.23 ± 0.16	1.35 ± 0.19	1.37 ± 0.14	1.37 ± 0.22
	ET-1 (pg/mL)	42.1 ± 5.52	43.0 ± 5.59	44.5 ± 5.27	41.3 ± 5.19	40.7 ± 4.60
	TNI (ng/mL)	0.10 ± 0.07	0.10 ± 0.08	0.11 ± 0.07	0.13 ± 0.08	0.12 ± 0.09

SF-28D (*n* = 5)	vWF (ng/mL)	1.16 ± 0.36	20.78 ± 1.82^*∗∗*††^	16.07 ± 1.99^*∗∗*††^	10.34 ± 1.55^*∗∗*††^	1.64 ± 0.35^*∗*^
	ET-1 (pg/mL)	41.2 ± 6.40	64.2 ± 3.39^*∗∗*††^	76.0 ± 8.00^*∗∗*††^	60.7 ± 6.9^*∗∗*††^	46.4 ± 3.06^†^
	TNI (ng/mL)	0.12 ± 0.09	0.47 ± 0.12^*∗∗*††^	5.37 ± 1.59^*∗∗*††^	1.36 ± 0.59^*∗∗*††^	0.46 ± 0.10^*∗∗*††^

All data showed with mean ± standard deviation. ^*∗*^ *P* < 0.05, ^*∗∗*^ *P* < 0.01 compared with baseline. ^†^ *P* < 0.05, ^††^ *P* < 0.01 compared with CON group.
